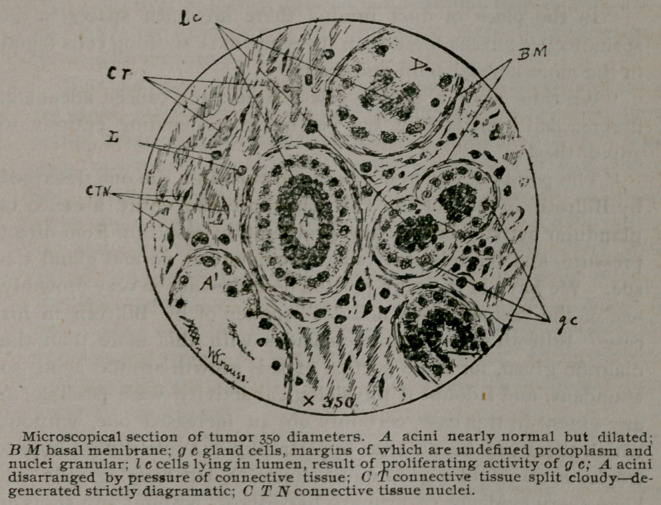# Acute Hyperthrophy of the Mammary Glands

**Published:** 1891-06

**Authors:** T. J. Crofford

**Affiliations:** Memphis, Tenn.


For Daniel's Texas Medical Journal.

### ACUTE HYPERTROPHY OF THE MAMMARY GLANDS.

BY T. J. CROFFORD, M. D., MEMPHIS, TENN.

THE FOLLOWING case is interesting on account of its rarity, on account of the rapidity of growth, and on account of the unusually large size to which the mammary organs have attained. So far as my investigations go with the literature at my command, it is the largest and most rapidly developed case of hypertrophy of the breasts on record.

Mollie H., aged fifteen years, was brought to me on the last of August, 1890. When she was but a few months past the age of fourteen this girl experienced her first menstruation.

At the time this came on she was suffering with la grippe and mumps. The menstruation at this time and subsequently ran the usual course. Prior to puberty, the breasts of this girl presented nothing unusual, but in a short while the mother noticed that they were unduly large, and ere long the enlargement amounted to a deformity. A physician was consulted; none of his remedies were of avail in checking the wild riotous growth which these organs had taken on.

When placed under my charge, although the measurements in inches as taken by Dr. A. B. Holder, of this city, were,

| | Right. | Left. |
|---|---|---|
| Circumference at base | 23 | 24½ |
| Circumference midway between base and nipple | 28½ | 31 |

| | | |
|---|---|---|
| Circumference from front of base over nipple and back to starting point | 32½ | 35½ |
| From sternal to axillary side of base over nipple | 27 | 29 |
| From base above to base below over nipple | 22½ | 24 |

yet I shrank at sacrificing the breast of a girl just budding into womanhood, and when I thought that she would be chagrined at not being like other girls, and above all be deprived of the highest boon of maternity should she ever become a mother, the revolt was complete, and they were told that although the authorities said there was nothing to be done in such cases except amputation, yet we would not amputate these organs without a thorough trial of compression. So with the assistance of the nurses and Dr. Holder, bandages were applied for two weeks, at the expiration of which time the breasts were quite as large as when we began. The operation was now determined upon. Lateral flaps were made, not wishing to risk anything on the cosmetic operations that have been devised. The bases were large, the organs were quite vascular, so it was impossible to avoid hemorrhage when the slightest cut was made into the organs. There was almost no cellular tissue and fat between the skin and the gland. No doubt these had been absorbed on account of the large and rapidly growing gland. Realizing that there would be quite a quantity of blood lost should the use of the knife be continued, the instrument was laid aside and the closely adherent skin was peeled off from the gland by the use of the handle of the knife and the fingers. In a similar manner the gland was removed from its attachment to the pectoral muscles. The sheath of the muscles was brought away with the gland.

There was one fact right here connected with the operation which surprised and impressed me; this was the absence of bleeding vessels at the base. Remembering the fact that the mammary glands have rather good-sized arteries from the internal mammary, from the intercostals and from the throacic branches of the axillary in their normal condition, then taking into consideration the greatly increased blood supply incident to this large and rapid growth, we were prepared to ligate some formidable vessels at the base, but to our surprise there was no bleeding beyond a moderate oozing and we were forced to the conclusion that these glands drew by far the greater part of their nutrition from the vessels entering through the skin.

In looking at the photograph the superficial veins can be plainly seen. Taking this fact into consideration, might it not be worthy of a trial early in the progress of a similar case to dissect up the skin and then replace it in its former position, hoping to change the abnormal nutrition and cause a shrinkage of the organ by breaking up its blood supply, without which it could not so lustily thrive?

She experienced a somewhat tardy healing, partly, I think, on account of the irritating and septic fluid which was considerable from the gland, and partly due to the want of sufficient circulation in the skin flaps after having been torn from the adherent organ.

Notwithstanding we left an excess of two inches of flaps there was a contraction in healing until it barely covered the wound. The right gland was amputated Sept. 16th, weighed 13 pounds. The left was removed Oct. 2nd, and weighed 11½ pounds. Two weeks later she returned home in good health and has since remained so.

Dr. Wm. Krauss, of this city, has kindly prepared a report of microscopical appearance of these organs, which is the most interesting part of the case and reads as follows:

"*Dr. T. J. Crofford, City:*

"The tumor sent me for examination is one of those rapidly growing circumscribed benign neoplasms which have been variously styled diffuse adenoma, acute fibro adenoma, acute diffuse hypertrophy, etc.

"Microscopically it appears like a fatty tumor, doughy to the touch but rather more nodular, with firm centres. On section it looks white, with very few vascular spots, soft in portions. The exuding juice consists of fatty and granular cells. Some portions are firm like collections of fibromata. Near the base of the tumor and a little to one side a pink mass the size of a walnut was found, differing from all the balance of the growth both in gross and microscopic appearance.

"Under a low power the tumor is seen to consist mostly of fibrous stroma without a fatty tissue, the gland tissue being in places normal, but everywhere pervaded by the growing fibrous matrix, showing every gradation from simple increase of stroma to complete destruction of gland, loose epithelial cells being imprisoned like in a very firm scirrhus. For the most part it looks like fibro-adenoma, the cells lying in open spaces, often arranged in concentric layers surrounded by a wall of firm fibrous tissue.

Microscopical section of tumor 350 diameters. *A* acini nearly normal but dilated; *B M* basal membrane; *g c* gland cells, margins of which are undefined protoplasm and nuclei granular; *l c* cells lying in lumen, result of proliferating activity of *g c*; *A* acini disarranged by pressure of connective tissue; *C T* connective tissue split cloudy—degenerated strictly diagramatic; *C T N* connective tissue nuclei.

"Under a high power the connective tissue can be seen to split roughly, the bundles interlacing, by-a-line or cloudy, with very few nuclei. The acini are in some places nearly normal, though apparently dilated and filled with deeply staining cells arranged in one or more layers. Numerous lymph channels pervade the mass, and here the process of formative tissue generation can be seen in all stages: Escaping corpuscles, undergoing mytosis, young connective tissue cells in the act of growing and elongating, etc.

"Osmic acid preparations show a few minute fat globules scattered through all the tissues.

"The macroscopically pink portion differs from the main mass principally in not having any normal gland tissue in it; the acimi are only masses of highly staining cells without any effort at agreement. The stroma is characterized by having a large number of nuclei, the connective tissue being embryonic in appearance, a few nuclei give the impression of being those of unstriated muscle, particularly around the epithelial collections which take the place of acini.

"In the place of duct lumina there are open spaces in the sprindleelled stroma filled with the same dark staining cells found in the more normal acini and ducts.

"We thus have a rapid growth simulating cancer, adenoma, fibroma and hypertrophy, but yet not corresponding entirely to any of these.

"The points of difference between this and the one described by Billroth in one of his two cases, are the entire absence of glandular activity beyond the proliferation resulting from direct pressure, and the relatively smaller amount of normal gland tissue. We have in the pink portion described above very probably one of the "sarcomatous nodules" spoken of by Billroth in his case. Billroth's description coincides with this more than the diagram given, for in no portion of this growth are the acini so abundant, and I doubt if physiological activity were possible to any extent in this case, certainly not an increased one, without which there can be no true hypertrophy. Acute diffuse hypertrophy is no doubt a good name clinically, but histologically we have every evidence of primary hyperinosis without any signs of irritation—round cell infiltration, the gland cell proliferation being secondary.

"The extreme coarseness and interlacement of the fibrous tissue stamps it as a neoplasm.

"The most remarkable point in the histology of these tumors is that they are in every respect diffuse as far as the mamma is concerned, but do not invade the surrounding tissues.

"Very respectfully,

"WM. KRAUSS."

## Figures and Tables

**Figure f1:**
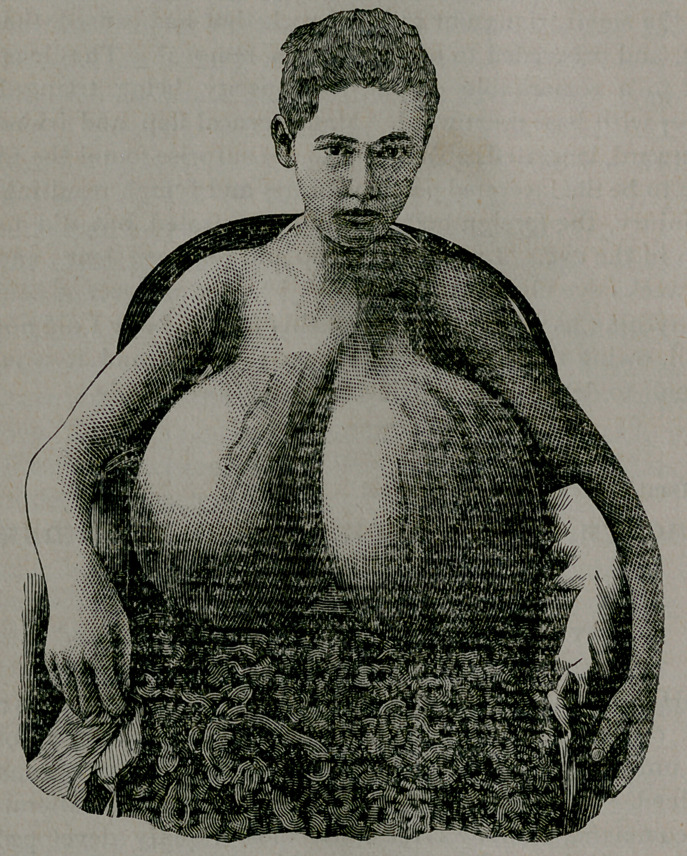


**Figure f2:**